# Biological informed graph neural network for tumor mutation burden prediction and immunotherapy-related pathway analysis in gastric cancer

**DOI:** 10.1016/j.csbj.2023.09.021

**Published:** 2023-09-22

**Authors:** Chuwei Liu, Arabella H. Wan, Heng Liang, Lei Sun, Jiarui Li, Ranran Yang, Qinghai Li, Ruibo Wu, Kunhua Hu, Yuedong Yang, Shirong Cai, Guohui Wan, Weiling He

**Affiliations:** a Department of Gastrointestinal Surgery, The First Affiliated Hospital, Sun Yat-Sen University, Guangzhou 510080, China; b Department of Pathology, The First Affiliated Hospital, Sun Yat-Sen University, Guangzhou 510080, China; c National-Local Joint Engineering Laboratory of Druggability and New Drug Evaluation, National Engineering Research Center for New Drug and Druggability (cultivation), Guangdong Province Key Laboratory of New Drug Design and Evaluation, School of Pharmaceutical Sciences, Sun Yat-Sen University, Guangzhou 510006, China; d Zhongshan School of Medicine, Sun Yat-Sen University, Guangzhou 510080, China; e School of Computer Science and Engineering, Sun Yat-sen University, Guangzhou 510006, China; f Department of Gastrointestinal Surgery, Xiang’an Hospital of Xiamen University, School of Medicine, Xiamen University, Xiamen, Fujian 361000, China

**Keywords:** Tumor mutation burden, Machine learning, Graph neural network, Immunotherapy, Gastric cancer

## Abstract

Tumor mutation burden (TMB) has emerged as an essential biomarker for assessing the efficacy of cancer immunotherapy. However, due to the inherent complexity of tumors, TMB is not always correlated with the responsiveness of immune checkpoint inhibitors (ICIs). Thus, refining the interpretation and contextualization of TMB is a requisite for enhancing clinical outcomes. In this study, we conducted a comprehensive investigation of the relationship between TMB and multi-omics data across 33 human cancer types. Our analysis revealed distinct biological changes associated with varying TMB statuses in STAD, COAD, and UCEC. While multi-omics data offer an opportunity to dissect the intricacies of tumors, extracting meaningful biological insights from such massive information remains a formidable challenge. To address this, we developed and implemented the PGLCN, a biologically informed graph neural network based on pathway interaction information. This model facilitates the stratification of patients into subgroups with distinct TMB statuses and enables the evaluation of driver biological processes through enhanced interpretability. By integrating multi-omics data for TMB prediction, our PGLCN model outperformed previous traditional machine learning methodologies, demonstrating superior TMB status prediction accuracy (STAD AUC: 0.976 ± 0.007; COAD AUC: 0.994 ± 0.007; UCEC AUC: 0.947 ± 0.023) and enhanced interpretability (BA-House: 1.0; BA-Community: 0.999; BA-Grid: 0.994; Tree-Cycles: 0.917; Tree-Grids: 0.867). Furthermore, the biological interpretability inherent to PGLCN identified the Toll-like receptor family and DNA repair pathways as potential combined biomarkers in conjunction with TMB status in gastric cancer. This finding suggests a potential synergistic targeting strategy with immunotherapy for gastric cancer, thus advancing the field of precision oncology.

## Highlights

• Distinct biological changes are identified in relation to varying TMB statuses through multi-omics data across 33 human cancer types.
• The PGLCN model, a biologically informed graph neural network, is developed to address the challenge of extracting meaningful biological insights from multi-omics data.
• PGLCN demonstrates superior performance in integrating multi-omics data for TMB prediction compared to previous machine learning methodologies.
• The Tol-like receptor family and DNA repair pathways are identified as potential combined biomarkers with TMB status for gastric cancer.

## Graphical abstract

**Figure:**
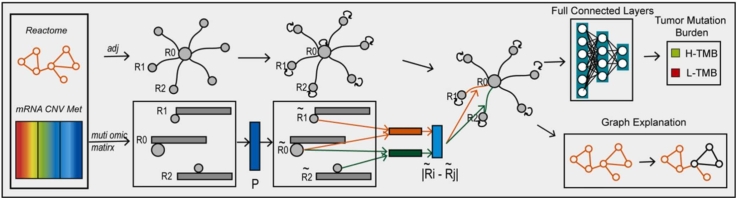
Interpretable biologically informed deep semi-graph neural network. The PGLCN consists three core modules: biological graph formation, neural network, and graph explanation. The biological graph formation module constructs a patient-specific biological graph based on biological priors and multi-omics data. The neural network module, consisting of graph learning layers, graph convolution layers, and fully connected layers, can learn novel pathway interaction patterns and predict tumor mutation burden (TMB) status. The graph explanation module generates feature and edge masks to identify essential biological subgraph structures, facilitating the generation of biological relevant hypotheses.

## 1 Introduction

Immune checkpoint therapy triggers the immune system, enabling immune cells to recognize and attack tumor cells. Since the U.S. Food and Drugs Administration (FDA) approved several immune checkpoint inhibitors (ICIs), immunotherapy has transformed the therapeutic landscape for numerous cancers [1], [2]. However, only a subset of patients achieve durable complete remission with these agents, underscoring the need for predictive biomarkers and combined therapy strategies. Tumor mutation burden (TMB), which measures the number of cancer mutations, has recently gained prominence. An accumulation of mutations can produce neoantigens, potentially aiding the immune system in tumor recognition [3], [4]. However, due to tumor complexity, TMB is not always correlated with ICI responsiveness of ICIs, necessitating TMB interpretation and contextualization refinement [5].

Recent advancements in multi-omics sequencing techniques have enhanced our understanding of tumor complexity [6]. Extracting biological insights from multi-omics information remains challenging, but predictive model interpretability in translational cancer genomics is essential for informing patient care and elucidating underlying biological processes [7]. Linear models, such as logistic regression, tend to offer higher interpretability with less accuracy. Deep neural network methods can handle complex structures and data dependencies with strong performance, but they are less interpretable.

Interpretability limitation hinder deep neural network applications in medicine [8]. Several interpretability approaches, such as Grad-CAM for image problems using global average pooling [9] and Lime for explaining black box classifiers [10], have improved our understanding of deep neural network functioning. GNNExplainer enhances graph neural network interpretability by identifying compact subgraph structures and crucial node features for predictions [11].

In this study, we introduce a novel biologically informed model, PGLCN, capable of stratifying cancer patients into subgroups with different TMB statuses and evaluating driver biological processes through model interpretability. We generated a multi-omics pathway matrix in a low-dimensional space using integrated multi-omics data, including mRNA expression, copy number variation (CNV), and DNA methylation [12], and established the pathway adjacency relationship based on the Reactome database. After modeling, we trained feature and edge masks as implemented in GNNExplainer to pinpoint essential pathway structures.

The availability of extensive molecular profiling data has facilitated the discovery of underlying biological mechanisms of different TMB statuses relevant to immunotherapy. By analyzing multi-omics data and TMB across 33 cancer types, we observed distinct biological changes between TMB statuses in STAD, COAD, and UCEC. The recent KEYNOTE-061 clinical trial identified a positive association between TMB and clinical outcomes in gastric cancer patients treated with pembrolizumab [13]. We further applied the PGLCN method to predict TMB status in gastric cancer patients. The PGLCN model exhibited superior predictive performance compared to previously established models. Importantly, through interpretability, PGLCN revealed that the the Toll-like receptor family and DNA repair pathway might function as a combined biomarker with TMB and could serve as a potential combined target strategy for gastric cancer immunotherapy [14].

## 2 Related works

In recent years, deep learning’s efficacy in meticulously identifying potential beneficiaries of immunotherapy has been underscored [15], [16]. Two major strategies have been delineated. The first encompasses predicting immunotherapy benefactors through multimodal clinical data assimilation, including CT/MRI imaging data [17], genomic sequences [18], [19], immunohistochemical data[20]. The second strategy incorporates prior knowledge in its modeling approach. Currently, salient biomarkers such as TMB[4], mismatch repair-deficient (MMR)[21], tumor-infiltrating lymphocyte (TIL)[22], MHCI [23], and PDL1[24] play a pivotal role in immunotherapy benefit prognostication. There is a burgeoning endeavor among researchers to amalgamate these biomarkers with clinical data for enhanced modeling [16]. For example, He et al. amalgamated TMB with CT imagery in a deep learning model tailored for advanced NSCLC, culminating in the formation of TMB radiomic indicators which exhibit a profound predictive acumen for ICI treatment responsiveness [25]. However, the inherent obscurity of deep learning models, attributed to their multifaceted parameter training, remains a challenge. Decipherable deep learning algorithms could augment precision in oncological immunotherapy research and unveil novel biological revelations.

Deep learning interpretability techniques can be taxonomically bifurcated into three paradigms based on their foundational principles: model-based interpretative methodologies, influence-based mathematical propagation, and transparent neural networks leveraging prior knowledge[26]. The former achieves interpretability via neuronal activity analysis [27] or by using attention mechanisms[28]. The influence-based mathematical propagation discerns pivotal input features either through the in-silico mutagenesis (ISM) forward propagation mechanism[29] or gradient-based backpropagation[12]. Lately, given the intricacies in interpreting deep neuronal functions, a proposition has emerged that emphasizes the encoding of transparent neural frameworks enriched with prior biological knowledge[7], [30].

Graph networks have shown unparalleled performance advantages across diverse biomedical fields in recent years[31], [32], [33], [34], [35], [36], [37]. For example, in juxtaposing genomic and histopathological data, Ding et al. demonstrated that spatially connected graph models enable accurate molecular profile predictions[32]. Within genomics, Webber et al. scrutinized the competency of graph neural networks in classifying multi-cancer types[37]; while in bio-electrical signal processing, Duong et al. applied these networks to adeptly categorize electrocardiogram outputs[34]. Yet, an in-depth exploration of the interpretability facets of graph networks in medical domains remains largely untapped. Graph neural structures rooted in prior knowledge hold promise in discovering critical biological regulatory networks.

## 3 Method

### 3.1 Data Collection

The Cancer Genome Atlas (TCGA) datasets were collected from the UCSC database (http://xena.ucsc.edu/)[38] and cBioPortal database (http://cbioportal.org)[39]. The multi-omics data (mRNA expression, CNV, and DNA methylation) and clinical information for cancer patients were obtained. Simple nucleotide data were acquired from TCGA GDC (https://portal.gdc.cancer.gov/repository). High tumor mutation burdens were defined as gene mutant rates > 10per million bases. The Synthetic Minority Over-Sampling Technique (SMOTE) was used to balance the data. Reactome datasets were collected from the Reactome pathway datasets (https://reactome.org/)[40], including complete information on pathways, pathways hierarchy relationship, and pathways gene sets. Immunotherapy datasets were collected from the tide database (http://tide.dfci.harvard.edu/download/)[41], [42], [43]. The Kim cohort (immune blockade therapy cohort of Gastric cancer) was obtained. Synthetic dataset construction encompassed the creation of five distinct node classification datasets. Initial graphs for BA-House and BA-Grid were instantiated based on the foundational Barabási–Albert (BA) schema, incorporating 300 nodes. A subsequent integration of 80 network motifs was carried out — specifically, the "house" five-node architecture for BA-House and a 3 × 3 grid configuration for BA-Grid appended to randomly selected nodes within the foundational graph. The BA-Community dataset amalgamates two BA-House graphs in its formulation. In the cases of Tree-cycle and Tree-Grid datasets, their foundational structure was an 8-tier balanced binary tree. Following this, 80 network motifs-encompassing a 6-node "cycle" structure for Tree-cycle and a recurring 3 × 3 grid motif for Tree-Grid were fused to randomly pinpointed nodes within the basic graph architecture [11].

### 3.2 PGLCN architecture overview

**Fig. 1:**
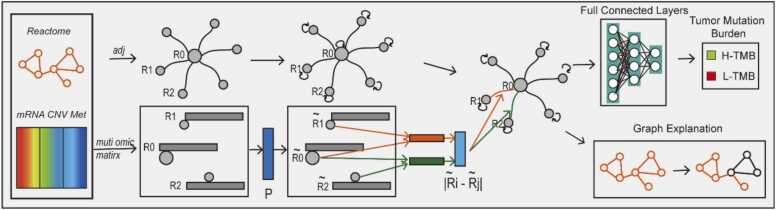
Interpretable biologically informed deep semi-graph neural network. The PGLCN consists three core modules: biological graph formation, neural network, and graph explanation. The biological graph formation module constructs a patient-specific biological graph based on biological priors and multi-omics data. The neural network module, consisting of graph learning layers, graph convolution layers, and fully connected layers, can learn novel pathway interaction patterns and predict tumor mutation burden (TMB) status. The graph explanation module generates feature and edge masks to identify essential biological subgraph structures, facilitating the generation of biological relevant hypotheses.

As illustrated in Fig. 1, PGLCN comprises three primary modules: biological graph formation, neural network, and graph explanation. For each patient, a biological graph is constructed where nodes represent specific pathways, node features depict multi-omics characteristics for each pathway, and node interactions signify relationships among pathways. The proposed architecture is employed to discover new pathway interaction patterns and extract features. Fully connected layers are utilized to map the distributed feature representation to the label space, enabling TMB status prediction. To identify crucial subgraphs associated with TMB states, an independent model is trained for model explanation, generating a feature mask and edge mask.

### 3.3 Construct biological graph

The hierarchy relationship of pathways was obtained from the Reactome database, and the adjacency matrix $A\in R^{p\times p}$ was constructed, where p represents the number of Reactome pathways. Direct interaction between different pathways were marked as 1, while no direct interaction was marked as 0. Diagonal elements were set to 1 to represent self-linking relationship within A.

Multi-omics features of each pathway were constructed using mRNA expression, CNV, and DNA methylation data, which were transformed to matrix $G,C,M\in R^{n\times r}$, where n and r represent the number of patients and genes, respetively. For each Reactome pathway p_i_, associated genes were extracted from matrices G, C, and M, generating intermediate matrices $G_{I}\;C_{I},M_{I}\in R^{n\times r_{i}}$, where $r_{i}$ represents the number of genes involved in pathway $p_{i}$. Principal component analysis (PCA) was applied to decompose matrices $G_{I}\;C_{I},$ and $M_{I}$ into uncorrelated components, yielding $Gp_{i},Cp_{i},Mp_{i}\in R^{n\times q}$, where q denontes the number of principal components (PCs). This processes was repeated for all Reactome pathways, yielding merged matrices $\mathrm{Gp},\mathrm{Cp},\mathrm{Mp}\in R^{n\times \mathrm{pq}}$, where p represents the number of Reactome pathways. A rearranged and combined matrix of the three matrices，$\mathrm{Kp}\in R^{p\times 3q}$, was input into the PGLCN model, where p represents the number of Reactome pathways, and columns represent the combined PCs of three omics types. For example, when q= 2, Kp is represented by a matrix with p x 6 elements, with the first and second columns originnating from mRNA expression, the third and fourth columns from CNV, and the fifth and sixth columns from DNA methylation.

### 3.4 Constructing the graph learning module of PGLCN

The fixed graph may not be an optimal structure for graph CNNs. Previous studies have proposed models such as GLCN, which integrates graph learning and graph convolution in a unified model to learn an optimal graph structure that best serves CNNs[44], [45]. Su et al. proposed a graph classification CNNs to identify the distant metastasis cases, which embedded graph learning modules to construct an optimal protein-protein interaction network that optimally learned the gene interaction strength[46]. Based on these works, we designed a semi-supervised graph classification CNNs, capable of optimally learning pathway interaction strength and predicting patients' TMB states.

As a complex interaction of different pathways mediates biological processes and cellular function, Elmarakeby et al. had built a biologically informed sparse connected network based on the hierarchy relationship of pathways[7]. In this study, we constructed a biological graph for each patient based on pathway relationships and multi-omics data, using the pathway multi-omics matrix $X=(x_{1},x_{2},{...x}_{n})\in R^{p\times q}$ and the adjacency matrix $A=(x_{1},x_{2},{...x}_{n})\in R^{p\times p}$, where p represents the number of pathways, n represents the number of patients, and q represents the number of principals. We built a nonnegative function $S_{\mathrm{ij}}=h(g_{i}-g_{j})$to establish the pairwise relationship of pathways optimally. The large dimension P of the pathway matrix reduces the efficacy of $h(g_{i}-g_{j})$ for a long weight vector needing to be trained. Dimension reduction was achieved via a low dimensional embedding network parameterized by a projection matrix $P\in R^{\mathrm{np}\times d},d<\mathrm{np}$. The graph learning was constructed in an individual layer.

(1) $$\tilde{g_{i}}=g_{i}P,\mathrm{for i}=1,2,......p$$

(2) $$S_{\mathrm{ij}}=h\left(\tilde{g_{i}}-\tilde{g_{j}}\right)=\frac{A_{\mathrm{ij}}\exp\left(\mathrm{ReLU}\left(a^{T}\left|\tilde{g_{i}}-\tilde{g_{j}}\right|\right)\right)}{\sum_{j=1}^{n}A_{\mathrm{ij}}\exp\left(\mathrm{ReLU}\left(a^{T}\left|\tilde{g_{i}}-\tilde{g_{j}}\right|\right)\right)}$$

Where ReLU(.) is an activation function to ensure the nonnegativity of $S_{\mathrm{ij}}$, and $a^{T}$ is the weight vector. The final softmax operation ensures that the learned graph S can satisfies the following properties:

(3) $$\sum_{j=1}^{n}S_{\mathrm{ij}}=1,S_{\mathrm{ij}}>0$$

The following loss function was used to optimize vector a and projection matrix P.

(4) $$L1=\sum_{i,j=1}^{n}\mathrm{||}\;g_{i}-g_{j}|{|}_{2}^{2}S_{\mathrm{ij}}+\gamma \mathrm{||S|}{|}_{F}^{2}$$

That is, a larger distance $\mathrm{||}g_{i}-g_{j}\mathrm{||}2$ encourages a smaller value $S_{\mathrm{ij}}$. The second term is a regularization used to control the sparsity of S.

### 3.5 Graph convolutional module of PGLCN

As shown in Fig. 1, PGLCN contains one graph learning layer, several convolution layers, and multiple fully connected layers. Since the graph S satisfies the properties: $\sum_{j=1}^{n}S_{\mathrm{ij}}=1,S_{\mathrm{ij}}>0$, the graph convolution layer was simplified as follow:

(5) $$X^{\left(k+1\right)}=\sigma (SX^{\left(k\right)}W^{\left(k\right)})$$

Where k = 0, 1, 2…k-1. $X^{\left(k+1\right)}$ denotes the output of activations in the (k + 1)-th layer. σ, denotes the activation function. $W^{\left(k\right)}$ is a trainable weight matrix. Specifically, $W^{\left(0\right)}{\in R}^{1\times g(0)}$ is an input-to-hidden trainable weight matrix, and $W^{\left(k\right)}\in {\;R}^{g(k-1)\times c}$ is a hidden-to-output trainable weight matrix, where c represents the class number.

The cross-entropy loss function was minimized to optimize the weight matrices used in convolution layers and fully connected layers.

(6) $$L2=-\sum_{i\in T}\sum_{j=1}^{2}Y_{\mathrm{ij}}\ln Z_{\mathrm{ij}}$$

Where $Z\in R^{n\times 2}$ represents the predicted label, $Y\in R^{n\times 2}$ represents the true label.

The entire architecture’s parameters were optimized using the combined loss function:

(7) L=L1+λL2

Where λ is a tradeoff parameter.

### 3.6 Cross-validation

A 5-fold cross-validation (CV) with five repeats was used to estimate the model. A stratified K fold was used to split the sample. 20% of samples were used as the test sample. The remaining data were further split into a training set (80%) and a validation set (20%). Accuracy, Recall, F1 score, AUC, and Precision were used to quantify the model. The model’s performance was compared with several well-established machine learning models, including L2 Logistic Regression, RBF Support Vector Machine, Linear Support Vector Machine, Random Forest, Adaptive Boosting, and Decision Tree.

### 3.7 Biological interpretation using GNNExplainer

GNNExplainer is a model-agnostic approach that explains GNNs’ prediction on graph-based machine learning tasks by learning a graph mask and a feature mask, selecting important subgraphs, and masking unimportant node features [11].

For a given biological graph G with feature Matrix $X=(x_{1},x_{2},...x_{n})\in {\mathbb{R}}^{n\times q}$ and adjcentmatrix $E=(x_{1},x_{2},...x_{n})\in {\mathbb{R}}^{p\times p}$, we trained a graph mask $M\in {\mathbb{R}}^{p\times p}$ and a feature mask $F\in {\mathbb{R}}^{n\times q}$ which is similar to GNNExplainer to calculate the sample level importance of pathways, where p represents the number of nodes and q represents the number of the genomic principal features. Our goal was to identify a subgraph $G_{s}$ with feature Matrix $X_{s}=X⊙\sigma (F)$ and adjcent matrix $E_{s}=E⊙\sigma (M）$, crucial for the PGLCN’s prediction $\hat{y}$. We utilized mutual information (MI) to select the most important structure and features.

(8) L1=MIY,Gs,Xs=HY−HY|G=Gs,X=XsGsmax

Given a trained PGLCN model Φ. MI quantifies the change in the probability of prediction $\hat{y}=\Phi (G,X)\;$when its feature matrix is limited to $X_{s}$ and its edge matrix limited to $E_{s}$. For the entropy term H(Y) is constant because Φ is fixed for a trained model, maximizing mutual information is equivalent to minimizing conditional entropy$\;H(\mathrm{Y|G}=G_{s},X=X_{s})$. that is:

(9) $$L_{1}=\min H\left(Y|,G=G_{s},X=X_{s}\right)=\min-E_{\mathrm{Y|}G_{s},X_{s}}\left[\log P_{\Phi }\left(Y|,{G=G}_{s},X=X_{s}\right)\right]$$

L2 and L3 were then used to regulate the entropy loss of graph mask M and feature mask F respectively.

(10) $$L_{2}=\theta *\mathrm{mean}(-M*\log(M)-(1-M)*\log(1-M))$$

(11) $$L_{3}=\theta *\mathrm{mean}(-F*\log(F)-(1-F)*\log(1-F))$$

Furthermore, L4 and L5 were used to limit the size of mask M and mask F, respectively.

(12) $$L_{4}=\theta *\mathrm{mean}\left(\sigma \left(M\right)\right)$$

(13) $$L_{5}=\theta *\mathrm{mean}(\sigma (F))$$

θ represents the weight for each loss function.

## 4 Results

### 4.1 Visualization of multi-omics biological features

**Fig. 2:**
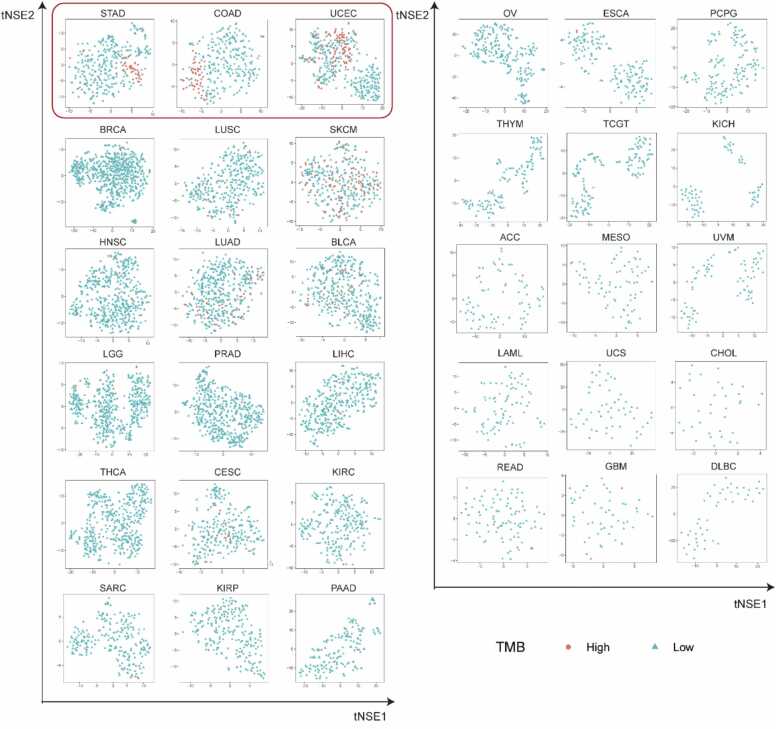
Visualization of multi-omics biological features. T-SNE visualization based on multi-omics feature matrices across 33 human cancer types. The multi-omics feature matrices consist of s rows (each row represents the same pathways) and six columns. Columns are formed with the first 2 PCs of each omic type (in the order of mRNA expression, CNV, and DNA methylation). High TMB is defined as gene mutant rates > 10 per million bases. Red dots represent patients with high TMB status, while blue dots represent patients with low TMB status.

We initially evaluated the biological variations between distinct TMB statuses across 33 human cancer types. Each patient’s multi-omics biological features matrix comprised s rows (corresponding to the same pathways) and 3q columns (with q representing the number of principal components, or PCs). In our model, q= 2, and the first 2PCs of each omic type (ordered as mRNA expression, CNV, and DNA methylation) constituted the columns. The t-Distributed Stochastic Neighbor Embedding (t-SNE) technique facilitates high-dimensional data mapping to lower dimensions while preserving local characteristics. Consequently, we employed t-SNE to visualize patients’ multi-omics biological features across cancer types (Fig. 2). Notably, distinct biological variations between different TMB statuses were observed in STAD, COAD, and UCEC, setting them apart from other cancer types. This finding suggests that patients with high TMB statuses exhibit conserved biological alterations in these three cancer types. Investigating the specific changes in biological processes may enhance TMB prediction accuracy and provide novel insights into immunotherapy strategies.

### 4.2 Model performance

**Fig. 3:**
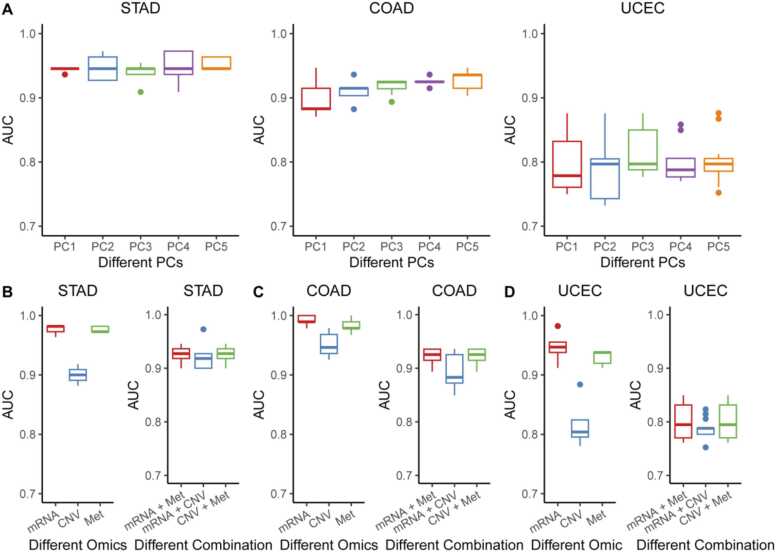
Performance comparison of PGLCN models. A. Evaluation of different sizes of multi-omics matrices using the top one principal component through five principal components for generating multi-omics matrices - PC, principal component; AUC, area under the curve. The upper and lower ends of the boxes represent the interquartile range of AUC values. The lines in the boxes indicate the median value, and black dots display outliers. B-D. Assessment of PGLCN performance using individual omics (mRNA Expression, CNV, and DNA methylation) or a combination of two omics (mRNA Expression + DNA methylation, mRNA Expression + CNV, CNV + DNA methylation). The upper and lower ends of the boxes represent the interquartile range of AUC values. The lines in the boxes indicate the median value, and black dots display outliers.

Subsequently, we trained the PGLCN model to classify high and low TMB groups in STAD, COAD, and UCEC. A 5-fold cross-validation (CV) scheme with 5 repeats was employed to assess the model's performance using various PC sizes (1−5) (Fig. 3A). With 2 PCs, the performance of PGLCN reached saturation, exhibiting an average AUC of 0.948 ± 0.019, 0.910 ± 0.017, and 0.791 ± 0.052 for STAD, COAD, and UCEC, respectively.

We then sought to determine the more informative omics for the model (Fig. 3B-D). Firstly, the performance of the model was accessed using individual omics. In STAD, the PGLCN model with each omic type yielded an average AUC of 0.976 ± 0.007, 0.900 ± 0.013, and 0.976 ± 0.004 for mRNA expression, CNV, and DNA methylation, respectively. Additionally, the PGLCN model with a combination of two omic types displayed an average AUC of 0.924 ± 0.015, 0.923 ± 0.028, 0.922 ± 0.026 for combinations of mRNA expression and DNA methylation, mRNA expression and CNV, and CNV and DNA methylation, respectively. Similar results were observed in COAD and UCEC. Collectively, these findings indicate that mRNA expression is more informative for the model.

### 4.3 Comparison with conventional machine learning models

**Fig. 4:**
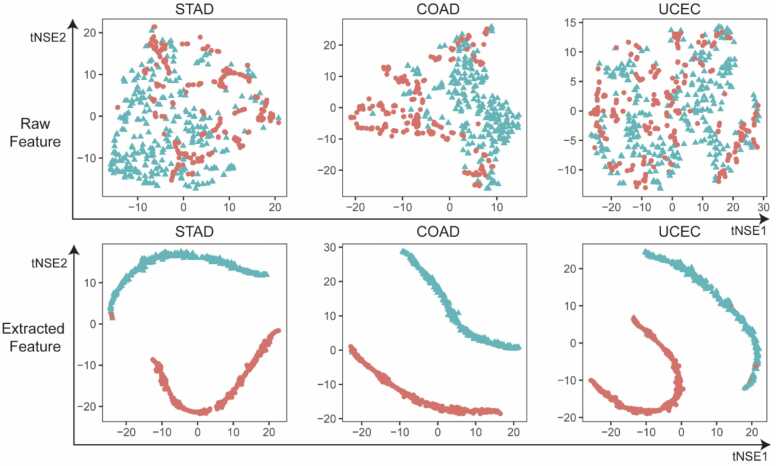
Visualization of features extracted from PGLCN. T-SNE method employed for visualization based on critical features and features extracted from PGLCN. High tumor mutation burden was defined as gene mutant rates > 10 per million bases. Red dots represent patients with high TMB status, while blue dots represent patients with low TMB status.

We employed the t-SNE method to investigate whether the features extracted from PGLCN’s final fully connected layer are more separable (Fig. 4). Following the graph convolution operation, the two labels exhibited highly separability. This result suggests that the features derived from PGLCN are discriminative and could potentially enhance prediction performance.

Furthermore, we compared the predictive performances of PGLCN with six distinct machine learning methods, including L2 Logistic Regression, RBF Support Vector Machine, Linear Support Vector Machine, Random Forest, Adaptive Boosting, and Decision Tree. As shown in Table 1, Table 2, Table 3, within the STAD dataset, PGLCN, alongside the Linear Support Vector Machine and Random Forest models, demonstrated superior predictive capacities compared to other methods. Significantly, PGLCN achieved the zenith in Recall metrics. In subsequent evaluations with the COAD and UCEC datasets, PGLCN consistently emerged as the top performer. Notably, PGLCN exhibited a smaller standard deviation, indicating greater stability compared to alternative approaches.

**Table 1:** Comparison of predictive performance of PGLCN with typical machine learning models in terms of area under the curve (AUC: mean six standard deviations) using 5-fold cross-validation on STAD dataset.

Methods	Accuracy	Recall	F1 score	AUC	Precision	Time (second)
L2 Logistic Regression	0.876 ± 0.027	0.989 ± 0.017	0.889 ± 0.021	0.876 ± 0.027	0.809 ± 0.037	0.147 ± 0.014
RBF Support Vector Machine	0.902 ± 0.039	0.804 ± 0.077	0.889 ± 0.046	0.902 ± 0.039	1 ± 0	1.581 ± 0.063
Linear Support Vector Machine	0.987 ± 0.011	1 ± 0	0.988 ± 0.011	0.987 ± 0.011	0.976 ± 0.021	0.516 ± 0.02
Random Forest	0.98 ± 0.013	0.975 ± 0.025	0.979 ± 0.014	0.98 ± 0.013	0.985 ± 0.013	0.887 ± 0.028
Adaptive Boosting	0.973 ± 0.019	0.975 ± 0.019	0.973 ± 0.018	0.973 ± 0.019	0.972 ± 0.032	12.028 ± 0.084
Decision Tree	0.931 ± 0.025	0.944 ± 0.038	0.932 ± 0.024	0.931 ± 0.025	0.922 ± 0.043	0.872 ± 0.044
PGLCN	**0.976 ± 0.007**	**0.985 ± 0.022**	**0.977 ± 0.008**	**0.976 ± 0.007**	**0.968 ± 0.013**	**45.978 ± 0.433**

**Table 2:** Comparison of predictive performance of PGLCN with typical machine learning models in terms of area under the curve (AUC: mean six standard deviations) using 5-fold cross-validation on COAD dataset.

Methods	Accuracy	Recall	F1 score	AUC	Precision	Time (second)
L2 Logistic Regression	0.911 ± 0.021	0.999 ± 0.004	0.918 ± 0.018	0.911 ± 0.022	0.85 ± 0.03	0.094 ± 0.018
RBF Support Vector Machine	0.861 ± 0.034	0.723 ± 0.068	0.837 ± 0.046	0.861 ± 0.034	1 ± 0	1.252 ± 0.055
Linear Support Vector Machine	0.983 ± 0.009	0.996 ± 0.009	0.983 ± 0.009	0.983 ± 0.009	0.971 ± 0.017	0.262 ± 0.021
Random Forest	0.983 ± 0.012	0.989 ± 0.012	0.984 ± 0.012	0.983 ± 0.012	0.979 ± 0.022	0.664 ± 0.02
Adaptive Boosting	0.947 ± 0.018	0.979 ± 0.019	0.948 ± 0.018	0.947 ± 0.018	0.92 ± 0.018	10.186 ± 0.069
Decision Tree	0.93 ± 0.019	0.931 ± 0.037	0.93 ± 0.018	0.929 ± 0.019	0.932 ± 0.046	0.638 ± 0.108
PGLCN	**0.994 ± 0.007**	**0.998 ± 0.006**	**0.994 ± 0.007**	**0.994 ± 0.007**	**0.989 ± 0.011**	**31.702 ± 11.002**

**Table 3:** Comparison of predictive performance of PGLCN with typical machine learning models in terms of area under the curve (AUC: mean six standard deviations) using 5-fold cross-validation on UCEC dataset.

Methods	Accuracy	Recall	F1 score	AUC	Precision	Time (second)
L2 Logistic Regression	0.86 ± 0.035	0.959 ± 0.025	0.873 ± 0.029	0.86 ± 0.035	0.802 ± 0.044	0.162 ± 0.023
RBF Support Vector Machine	0.823 ± 0.017	0.646 ± 0.032	0.784 ± 0.023	0.823 ± 0.016	1 ± 0	1.743 ± 0.03
Linear Support Vector Machine	0.933 ± 0.012	0.975 ± 0.025	0.935 ± 0.012	0.933 ± 0.012	0.899 ± 0.021	0.692 ± 0.02
Random Forest	0.905 ± 0.032	0.911 ± 0.042	0.906 ± 0.033	0.905 ± 0.032	0.902 ± 0.041	1.147 ± 0.043
Adaptive Boosting	0.851 ± 0.02	0.894 ± 0.049	0.857 ± 0.018	0.851 ± 0.02	0.827 ± 0.044	12.305 ± 0.036
Decision Tree	0.797 ± 0.028	0.814 ± 0.042	0.801 ± 0.021	0.798 ± 0.028	0.794 ± 0.057	1.22 ± 0.123
PGLCN	**0.947 ± 0.023**	**0.968 ± 0.021**	**0.948 ± 0.023**	**0.947 ± 0.023**	**0.93 ± 0.034**	**46.024 ± 0.187**

### 4.4 Model interpretability

**Fig. 5:**
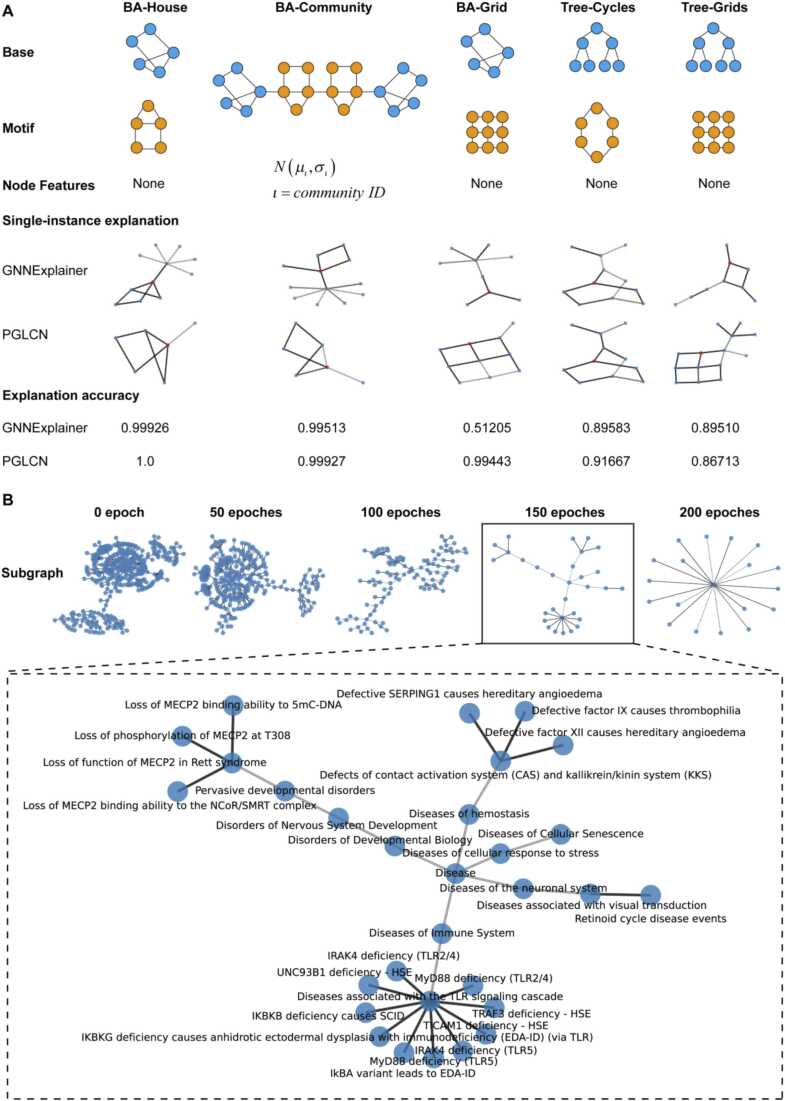
Interpretative Performance of the PGLCN Model. A. The PGLCN's interpretability was assessed using the GNNEXPLAINER model as a reference on five synthetic datasets featuring different motifs. The explanation accuracy was gauged using the AUC score. B. Utilizing PGLCN to unveil the pathway map linked to TMB status in gastric cancer. With the progression of training epochs, PGLCN begins to emphasize smaller subgraphs. By the 175th epoch, the model shifts its attention to disease-centric pathway subgraphs, particularly those associated with the immune system.

To examine the interpretability of the PGLCN model, we constructed five distinct datasets, positioning GNNEXPLAINER as a reference baseline. Our findings underscored the heightened interpretability of PGLCN across the BA-House, BA-Community, BA-Grid, and Tree-Cycles datasets. While PGLCN manifested a marginal decline in accuracy vis-à-vis GNNEXPLAINER within the Trees-Grids dataset, it exhibited a more pronounced aptitude in discerning pivotal subgraph configurations (Fig. 5A).

These empirical observations intimate that PGLCN's semi-supervised learning stratum augments the model's interpretability. Proceeding with this insight, we leveraged PGLCN's interpretability module to unearth salient biological network architectures germane to the TMB status in gastric cancer. Intriguingly, with successive training iterations, PGLCN adeptly recognized biological subgraphs of varying magnitudes. By the 200th training epoch, PGLCN's learning trajectory approached saturation. On analyzing the data from the 150th epoch, it became evident that PGLCN displayed a predilection towards immune-centric disease pathways, with a focus on Toll-like receptors (TLRs) signaling-associated pathways (Fig. 5B). These revelations not only insinuate the potential of the TLR family as a prognostic metric for combinatorial immunotherapy of TMB but also posit it as a prospective therapeutic target for tailored tumor-immune interventions across diverse TMB spectrums.

### 4.5 Interplay among the TLR family, gastric cancer tumor microenvironment, and TMB

TLRs represent a crucial category of protein molecules involved in mediating innate immunity. These receptors specialize in recognizing evolutionarily conserved molecular structures derived from microorganisms. Out of the 11 identified TLR family members, 10 play pivotal roles in human immune responses, with certain members residing on the cellular surface and others localized within endosomal or lysosomal compartments. Recent study has associated TLRs with facets of tumorigenesis, including immune suppression, cell apoptosis, and immune system activation [47].

**Fig. 6:**
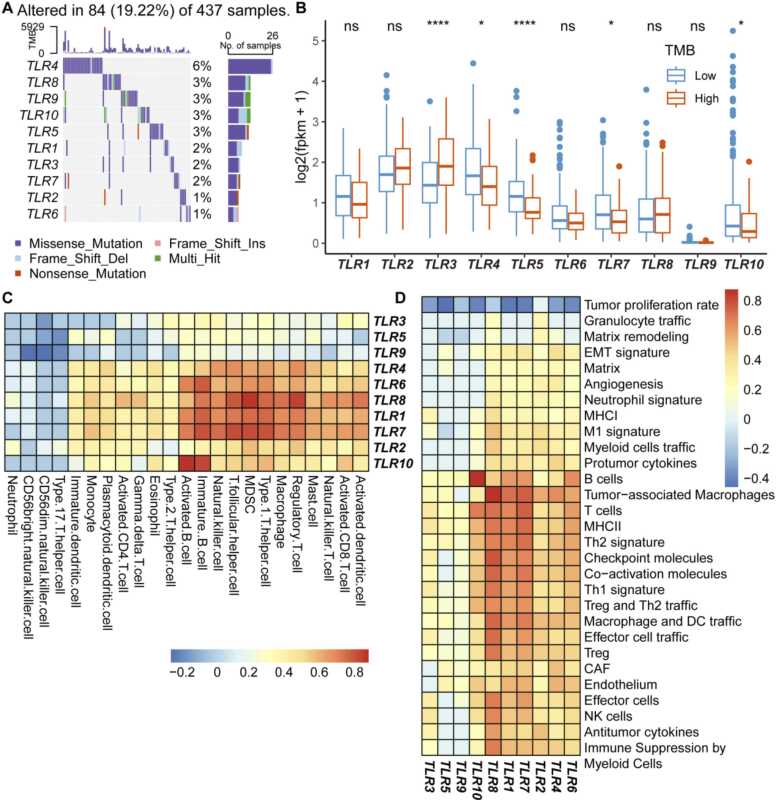
Interplay among TLRs, TMB, and the tumor microenvironment. A. Mutation prevalence of 10 Toll-like receptors across 437 gastric cancer patients from the TCGA-STAD cohort. Each column indicates an individual patient. B. Differential expression of the 10 Toll-like receptors among high and low TMB groups. High TMB is depicted in orange, while low TMB is in blue. Asterisks denote statistical significance (*P < 0.05; **P < 0.01; ***P < 0.001). C. Correlation heatmap illustrating the association between the 10 Toll-like receptors and the degree of tumor immune cell infiltration. Blue denotes a negative correlation, and red indicates a positive correlation. D. Correlation heatmap demonstrating the connection between the 10 Toll-like receptors and the constituents of the tumor immune microenvironment. Blue highlights a negative relationship, while red signifies a positive one.

Our data indicate mutations in members of the TLR family in approximately 19.22% of gastric cancer patients. Among these, TLR4 manifests the highest mutation prevalence, constituting 6%. In contrast, TLR2 and TLR6 mutations are relatively infrequent, representing a mere 1% (Fig. 6A). Subsequent analysis of TLR expression patterns across varying TMB statuses revealed an elevated expression of TLR3 in patients with a heightened TMB. Conversely, TLR4, TLR5, TLR7, and TLR10 expressions were decreased in high TMB tumors (Fig. 6B). Employing the ssGSEA algorithm, we discerned the degree of tumor immune cell infiltration and discerned tumor microenvironment characteristics. Intriguingly, TLR4, TLR6, TLR8, TLR1, TLR2, and TLR10 exhibited positive correlations with functional immune cell infiltration intensity. Notably, the inverse relationship these TLR receptors share with tumor proliferative dynamics (Fig. 6C-D).

As reported, TLRs function as conduits linking innate and adaptive immune cascades. By instigating cytokine release, they can potentiate cytotoxic T cells, thereby culminating in tumor annihilation and tumor burden diminution. These revelations underscore the potential of these TLRs as formidable therapeutic targets for combined tumor-immune treatments, specifically catering to diverse TMB profiles [48], [49].

### 4.6 Integrated analysis with the immunotherapy dataset

**Fig. 7:**
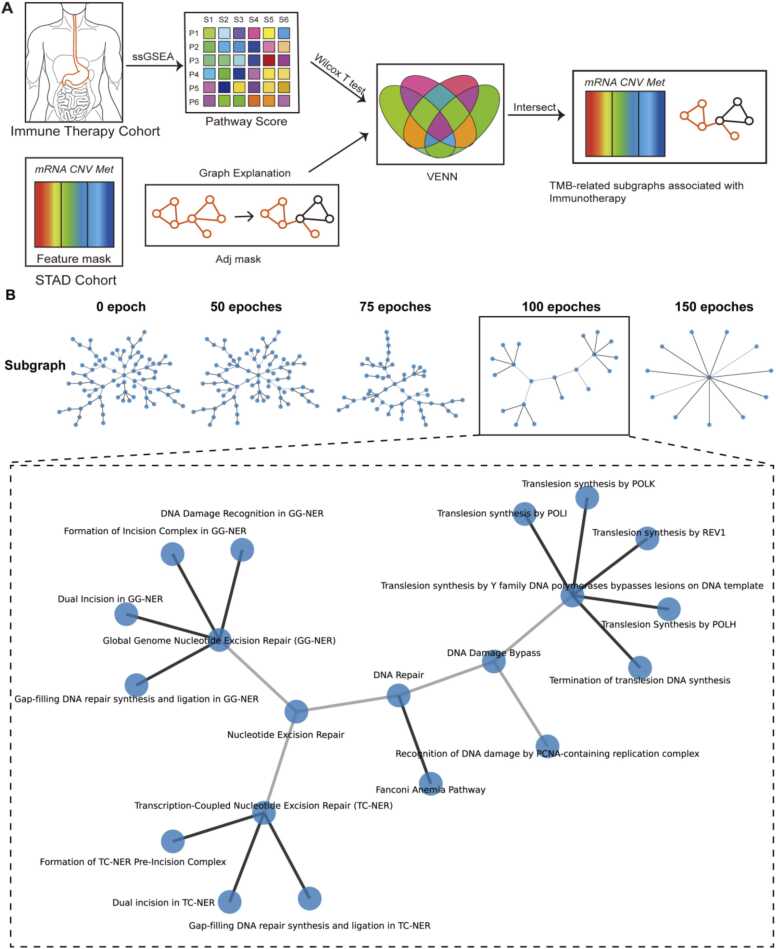
Integrative analysis incorporating the immunotherapy cohort. A. Workflow delineating the combined analysis utilizing the PGLCN model in conjunction with the immunotherapy dataset. B. Application of the PGLCN model to uncover pathway subgraphs associated with immunotherapy efficacy and TMB in gastric cancer. With the progression of training epochs, PGLCN emphasizes smaller subgraphs. Specifically, at the 100th epoch, the model highlights the Nucleotide Excision Repair pathway subgraph.

To discern the biological changes associated with TMB status that hold potential relevance for tumor immunotherapy, we employed the ssGSEA algorithm. This facilitated the calculation of pathway scores within the gastric cancer immunotherapy dataset. Utilizing the Wilcoxon T-test, we rigorously assessed the pathways bearing significance to immunotherapeutic efficacy. Subsequent integration with the graphical interpretations of PGLCN enabled the extraction of pertinent subgraph structures. Notably, PGLCN prominently highlighted the Nucleotide Excision Repair-associated pathway (Fig. 7A-B).

These observations resonate robustly with established biological knowledge. Elevated TMBs are inherently linked to an increased neoantigen presence, fostering enhanced lymphocyte infiltration. Concurrently, patients manifesting DNA repair deficiencies tend to exhibit a heightened proportion of somatic mutations. Such correlations intimate that the amalgamation of a high mutation load with tumor repair-associated pathways might serve as composite indicators, prognosticating the efficacy of tumor immunotherapy interventions [15], [50], [51], [52].

## 5 Discussion

Immune checkpoint blockage therapy can trigger patients' immune systems, leading to durable response. However, this therapy only benefits a small subset of patients, highlighting the need for predictive biomarkers and combination therapy strategies [1], [2]. While tumor mutation burden (TMB) is considered as a valuable biomarker for identifying potential responders to immune checkpoint inhibitors (ICIs), TMB does not always correlate with ICI responsiveness due to tumor complexity [4]. Investigating the underlying biological mechanisms and refining the contextualization of TMB is required.

Multi-omic sequencing provides insights into the genomic characterization of tumors, aiding in dissecting tumor complexity and playing a significant role in clinical impact. Genomic analysis may help refine the contextualization of TMB and contribute to precision immunotherapy [53], [54]. Furthermore, integrated genomics outperforms single-omics analysis [55], [56]. However, extracting biologically meaningful insight from such data remains challenging.

In this study, we leverage the high accuracy of neural networks and recent advancements in model interpretability to design a biologically informed graph neural network-PGLCN-for integrated multi-omics data analysis. PGLCN provides a framework for integrating hierarchical prior knowledge and multi-omics data, which previously required disparate statistical approaches. Consequently, PGLCN constructs a unique biological graph for each patient, enabling TMB status prediction based on specific biological graphs and identification of critical subgraph structures. Among 33 human cancer types, we observed distinct genomic changes between different TMB statuses in STAD, COAD, and UCEC, indicating the presence of shared biological changes. Identifying these biological changes helps enhance understanding of the underlying mechanisms associated with TMB status.

A recent clinical trial, KEYNOTE-061, identified a positive association between TMB and clinical outcomes in gastric cancer patients treated with pembrolizumab [13]. We further applied PGLCN to predict TMB status in STAD patients. PGLCN accurately predicted TMB statuses based on patients' biological graph profiles and identified crucial biological structures involved in regulating genomic instability. Visualizing significant sub-structures of biological graphs facilitates the development of hypotheses regarding the underlying biological processes involved in cancer TMB status. Moreover, we assessed PGLCN with a gastric cancer immunotherapy cohort [43]. Notably, our analyses pinpointed a strong association between the TLRs-related signaling graph and the TMB status in gastric cancer patients. Functioning as key conduits linking innate and adaptive immunity, TLRs have emerged as promising therapeutic targets in oncology, particularly in tumor immunotherapy [47], [48], [49]. This underscores the potential of TLRs as tailored therapeutic targets in tumor-immune synergistic treatments across varied TMB statuses. In a parallel inquiry integrating the PGLCN model with the immunotherapy dataset, there was a pronounced emphasis by the PGLCN on tumor repair-associated pathways. This alignment echoes prevailing biological understanding that patients with compromised DNA repair mechanisms tend to manifest elevated proportions of somatic mutations. Such observations reinforce the proposition that a synergy between high mutation loads and tumor repair-centric pathways might serve as robust combined indicators forecasting the therapeutic success of tumor immunotherapy [15], [50], [51], [52].

Although PGLCN provides a biologically informed framework for cancer genomic discovery, the model requires training before application. As with all deep learning models, hyperparameters used to train the model significantly influence the model’s performance. Additionally, PGLCN builds the biological graph structure based on biological priors, making the model’s application to different tasks dependent on these priors.

In conclusion, PGLCN, a biologically informed graph neural network, can accurately classify gastric cancer patients with different TMB statuses. Identifying important subgraph structures through random masking facilitates biological hypothesis generation. Combined analysis with the immunotherapy cohort highlights the critical role of NOTCH signaling in both tumor TMB status adaptation and tumor immunotherapy.

## Statement

During the preparation of this work, the authors declared no use any generative AI and AI-assisted technologies to generate any data of this work.

## Code availability

The data and source code generated in this study was deposited in GitHub [https://github.com/liuchuwei/PGLCN], which was implemented using Python, and Pytorch.

## Funding

This work was supported in part by grants from the 10.13039/501100001809National Natural Science Foundation of China (82122069, 82073869, 82022037); National Key Research and Development Plan (2022YFC3401000); Guangdong Basic and Applied Basic Research Foundation (2021B1515020004, 2021B1515230009); Guangdong Provincial Key Laboratory of New Drug Design and Evaluation (2020B1212060034); Key Research and Development Plan of Guangdong Province (2020B0101030006); the 10.13039/501100012226Fundamental Research Funds for the Central Universities (23yxqntd001).

## CRediT authorship contribution statement

GW and CL conceived the idea and designed the experiments. CL, AHW, HL, LS, JL, RY and QL collected and analyzed data; CL performed model establishment and bioinformatics analysis. KH, YY, SC, WH and GW provided administration and supervision. GW and CL wrote the manuscript. All authors were involved in final approval of the submitted and published versions.

## Declaration of Competing Interest

The authors declare that they have no known competing financial interests or personal relationships that could have appeared to influence the work reported in this paper.
